# Patients Admitted to Three Spanish Intensive Care Units for Poisoning: Type of Poisoning, Mortality, and Functioning of Prognostic Scores Commonly Used

**DOI:** 10.1155/2017/5261264

**Published:** 2017-03-28

**Authors:** María Esther Banderas-Bravo, Maria Dolores Arias-Verdú, Ines Macías-Guarasa, Eduardo Aguilar-Alonso, Encarnación Castillo-Lorente, Lucia Pérez-Costillas, Raquel Gutierrez-Rodriguez, Guillermo Quesada-García, Ricardo Rivera-Fernández

**Affiliations:** ^1^Intensive Care Unit, Regional University Hospital, Málaga, Spain; ^2^Intensive Care Unit, Infanta Margarita Hospital, Cabra, Córdoba, Spain; ^3^Intensive Care Unit, Neurotraumatology Hospital, Jaén, Spain; ^4^Psychiatric Department, Regional University Hospital, Málaga, Spain; ^5^Intensive Care Unit, Serrania Hospital, Ronda, Málaga, Spain

## Abstract

*Objectives*. To evaluate the gravity and mortality of those patients admitted to the intensive care unit for poisoning. Also, the applicability and predicted capacity of prognostic scales most frequently used in ICU must be evaluated.* Methods*. Multicentre study between 2008 and 2013 on all patients admitted for poisoning.* Results*. The results are from 119 patients. The causes of poisoning were medication, 92 patients (77.3%), caustics, 11 (9.2%), and alcohol, 20 (16,8%). 78.3% attempted suicides. Mean age was 44.42 ± 13.85 years. 72.5% had a Glasgow Coma Scale (GCS) ≤8 points. The ICU mortality was 5.9% and the hospital mortality was 6.7%. The mortality from caustic poisoning was 54.5%, and it was 1.9% for noncaustic poisoning (*p* < 0.001). After adjusting for SAPS-3 (OR: 1.19 (1.02–1.39)) the mortality of patients who had ingested caustics was far higher than the rest (OR: 560.34 (11.64–26973.83)). There was considerable discrepancy between mortality predicted by SAPS-3 (26.8%) and observed (6.7%) (Hosmer-Lemeshow test: *H* = 35.10; *p* < 0.001). The APACHE-II (7,57%) and APACHE-III (8,15%) were no discrepancies.* Conclusions*. Admission to ICU for poisoning is rare in our country. Medication is the most frequent cause, but mortality of caustic poisoning is higher. APACHE-II and APACHE-III provide adequate predictions about mortality, while SAPS-3 tends to overestimate.

## 1. Introduction

The prognosis and course of patients admitted to the intensive care unit (ICU) due to poisoning have not been widely studied in modern times, and there is, therefore, little written on the subject. This is an unusual pathology if we compare it to others which lead to admission to ICU, and perhaps this is why there are few studies in this respect [1]. Equally, there are few multicentre studies [2–4] providing databases with which to compare and contrast results.

In 1992, Nogué et al. [5] published the results of three multicentre studies carried out in Spain in 1980, 1987, and 1990. They included a total of 596 patients and observed the tendencies and changes that occurred during that decade. They found that only 5% of intoxicated patients treated in emergency departments required admission to the ICU. They also observed that the majority of patients were young (a mean age of 36) and largely male. The most common cause of poisoning was through the use of psychotropic drugs (mainly benzodiazepines and tricyclic antidepressants) for suicidal purposes. Mortality fluctuated between 6.4% and 9% and was significantly higher in cases of poisoning through drug abuse.

Being a pathology which generally affects younger people, we consider that poisoning carries special interest because it entails a high number of Potential Years of Life Lost. In 2005, Apellániz and Manzanaro [6] analysed data relating to mortality through poisoning from the Basque Registrar of Deaths* (Registro de Mortalidad de la Comunidad Autónoma Vasca)* between the years 1986 and 2001 and noted that there were 1,207 deaths due to poisoning during this period (0.45% of all deaths, with a gross mean of 3.58 deaths per 100,000 inhabitants), which mainly affected people under the age of 40 (65.45% of deaths occurring among people aged between 20 and 39). The mean Potential Years of Life Lost were 2,226.33/year, for a rate of 1.12/1000. However, being rare and having a low ICU and hospital mortality rate, within the field of critical care, death due to poisoning has become a somewhat forgotten pathology.

A study was recently published in 2014 on the subject of mortality and long-term survival of patients with acute poisoning admitted to the hospital in the Netherlands. This study observed that both ICU mortality and hospital mortality were low (1.2% and 2.1%, resp.), and the same was found two years after ICU admission (contrasting with other pathologies which require intensive care). The study observed differences in subgroups among patients according to the substances consumed but found no statistically significant differences [7]. Similarly, Liisanantti et al. [8] observed that poisoning represented 4.5% of all admissions to ICU, and the mean length of the ICU stay was very short (less than two days, with a mean length of ICU stay of 32.1 hours). Hospital mortality was 2.3%. However, upon admission, these patients had an APACHE-II score of 14.4 and a SOFA score of 4.8. These values suggest a higher severity compared with the low observed mortality rate. So, for this reason, we think that the prognostic scales we generally use in intensive care are not appropriate for determining the prognosis and course of patients with acute poisoning admitted to the ICU, and they do not provide good information about the risk of death to the families and medical staff.

We have recently published the results of a multicentre study evaluating the new version of SAPS-3 and APACHE-III in Spain [9, 10]. In this report, we included a very small number of patients suffering from poisoning. The objective was to evaluate patients with poisoning admitted to the ICU, their severity and mortality, and how well the prognostic scores SAPS-3, APACHE-II, and APACHE-III work with this group of patients.

## 2. Methods

This was a multicentre, observational study carried out from January 2008 to March 2013, including adult, critical patients with acute poisoning admitted consecutively to the Intensive Care Units of Carlos Haya Hospital in Málaga (hospital with 1000 beds, ICU with 40 beds), Infanta Margarita Hospital in Cabra, Córdoba (hospital with 258 beds, ICU with 11 beds), and Neurotraumatology Hospital in Jaén (hospital with 180 beds, ICU with 10 beds). Patients who were intoxicated but were admitted for other causes (brain trauma, polytrauma associated with motor vehicle accidents, etc.) were not included in the study.

The same data collection protocol was followed in each of the participating hospitals, including demographic variables, associated comorbidities, previous admission location (emergency, ICU, and ward), Glasgow coma score on admission, worst Glasgow score recorded during ICU stay, pupillary changes, the need for mechanical ventilation upon admission, and all the physiological and analytical variables taken one hour before, one hour after, and 24 hours after admission to ICU, required for the SAPS-3 prognosis system calculation [11, 12] and the diagnostic classification by APACHE-IV [13]. The poisoning subgroups based on the APACHE-IV reasons for admission were the following: (1) alcohol, (2) analgesics, (3) antidepressants, (4) street drugs, (5) sedatives, (6) poisoning (carbon monoxide, arsenic, and cyanide), (7) other toxic substances (caustics), and (8) combinations. This protocol also included the APACHE-II, APACHE-III, and SOFA scores [14–16] upon admission as well as the length of the ICU stay, the length of hospital stay, ICU mortality, and hospital mortality.

The data were entered prospectively into a database and were analysed retrospectively. The data were collected by physician assistants in the ICU who treat the patients daily.

This study was evaluated and approved by each hospital's ethics committee.

Statistical analysis was carried out using the PSPP statistical package for Windows. The results are expressed as mean ± standard deviation and median with interquartile range for quantitative variables and as absolute frequency and proportions for qualitative variables. For statistical analysis, we used Wilcoxon test and the chi-square test and for the multivariable analysis we carried out a multiple logistic regression and stepwise variable selection method. We used the Hosmer-Lemeshow [17] test to analyse the correlation between predicted mortality by the different prognostic scores and the observed mortality. *p* < 0.05 was considered statistically significant.

## 3. Results

During the period of the study, 19590 patients were admitted to the ICU of the three hospitals (15749 patients to the ICU of Carlos Haya Hospital, 1953 patients to the ICU of Infanta Margarita Hospital, and 1888 patients to the ICU of Neurotraumatology Hospital in Jaén).

In the same period, only 119 patients were admitted to the ICU for poisoning, that is, 76 from Carlos Haya Hospital, 21 from Infanta Margarita Hospital, and 22 from the Neurotraumatology Hospital in Jaén. The reasons for admission to ICU were poisoning due to medication in 92 patients (77.3%), alcohol in 20 (16.8%), and caustics in 11 (9.2%).

Within the group of medication-related cases, there were cases of poisoning from psychotropic drugs. Of these, 45.4% (54 patients) had taken benzodiazepines, 26.9% (32 patients) had taken tricyclic antidepressants, and 11.8% (14 patients) had taken antipsychotics or neuroleptic drugs. There were 10.1% (12 patients) intoxicated with paracetamol and 13.4% (16 patients) intoxicated with drugs of abuse (6.7% (8 patients) with cocaine and 6.7% (8 patients) with opiates). There were 46.2% (55 patients) suffering from poly-poisoning.

The main characteristics of the study sample are shown in Table 1. The mean age of the patients was 44.42 ± 13.85 years. Upon admission, they presented an APACHE-II score of 16.29 ± 7.17 points and an APACHE-III score of 47.68 ± 26.33 points, and severity evaluated by SAPS-3 was 54.17 ± 11.33 points. Mortality predicted by SAPS-3 was 26.98% according to the general equation and 27.78% for our geographical area.

The mean Glasgow Coma Scale (GCS) upon ICU admission was 8.39 ± 4.51 (72.5% of patients had a GCS ≤ 8). The majority of patients, 69.7%, required mechanical ventilation upon admission. 78.3% of cases attempted suicide.

The length of ICU stay was 5.73 ± 7.77 days and median was 2 days (interquartile range: 3 to 4). ICU mortality was 5.9% and hospital mortality was 6.7%. Those patients who died in hospital were older, had higher scores on the APACHE-II and APACHE-III scales, and had a higher probability of death according to these scores. The relationship between mortality and the different variables is shown in Tables 2(a) and 2(b).

Mortality was associated with the poisoning etiology (Table 2(b)); the mortality of the 11 patients admitted for the ingestion of caustics was 54.5%, compared with 1.9% in cases of noncaustic poisoning (*p* < 0.001). The mortality of the 92 patients admitted for the ingestion of drugs was 1.1%, while that of the other 27 patients was 9.9% (*p* < 0.001). The mortality of the 20 patients admitted for the ingestion of alcohol was 0%, while the mortality of the other 99 was 8.1% (*p* = 0.188).

Those patients who had ingested caustics, as well as presenting a higher rate of mortality, were also older and had better Glasgow scores on admission and higher APACHE-II and APACHE-III scores (Table 3).

In order to determine the existence of an independent relationship between hospital mortality and the ingestion of caustics, we carried out a logistical regression analysis. After adjusting for SAPS-3 (OR: 1.19 (1.02–1.39)), the mortality of patients who had ingested caustics was far higher than the rest (OR: 560.34 (11.64–26973.83)). Drug-induced and alcoholic poisoning variables were not included in the model due to the lack of statistical significance.

**Figure 1:**
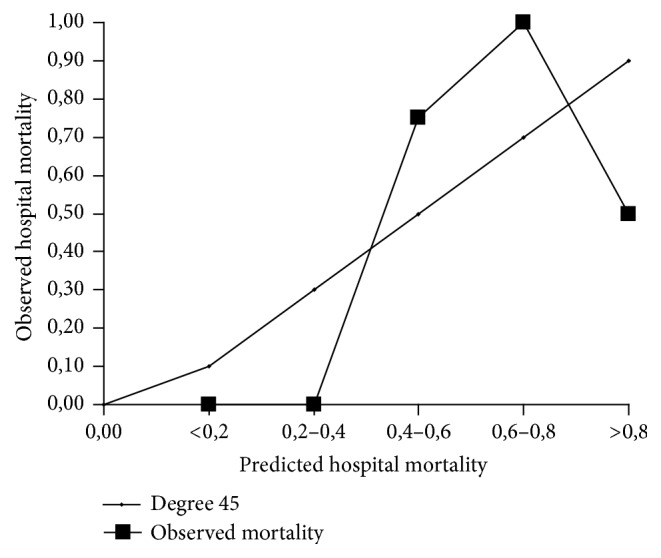
Predicted versus observed hospital mortality for APACHE-II model.

Finally, we assessed the agreement between observed mortality and predicted mortality using the three prognostic systems mentioned earlier (SAPS-3, APACHE-II, and APACHE-III) using the Hosmer-Lemeshow test. Thus, in the case of SAPS-3 (the general equation), we divided the population into various subgroups according to whether predicted mortality was below 20%, between 20 and 40%, between 40 and 60%, between 60 and 80%, or above 80%. Predicted mortality was, respectively, 11%, 29%, 47%, 68%, and 86%. The observed mortality was 6.6%, 2.2%, 7.7%, 20%, and 12.5%, respectively. So, there was a large discrepancy between predicted and observed mortality (*H* = 35.10), and this difference was statistically significant (*p* < 0.001) (Table 4(a)). Using the equation for our geographical area, there were similar discrepancies (*H* = 36.47; *p* < 0.001) (Table 4(b)). However, on analysing the agreement between observed mortality and predicted mortality using APACHE-II and APACHE-III, statistically significant differences were not found using the Hosmer-Lemeshow test (Tables 5(a) and 5(b), Figure 1). The predicted mortality was 7.57% using APACHE-II and 8.15% using APACHE-III, whereas the observed mortality was 6.7%, as stated above.

## 4. Discussion

This study showed that ICU admission by poisoning is infrequent. In a high percentage of cases, patients present with altered levels of consciousness, as indicated by the low Glasgow Coma Scale scores upon admission. This explains the high rate of initial mechanical ventilation. Severity according to SAPS-3 was high, but observed mortality was far lower than predicted, as opposed to what we observed with the APACHE-II and APACHE-III systems, which made adequate predictions about the probability of death in these patients. The mortality of patients admitted due to ingesting caustics was far higher than that of the other intoxicated patients.

The number of patients with acute poisoning admitted to the ICU was low, as our study shows. During the five years of the study, only 119 cases were recorded in three hospitals in Andalusia, with two of these being tertiary hospitals (*Hospital Regional Universitario Carlos Haya* in Málaga and* Hospital Neurotraumatológico* in Jaén) and one specialist hospital (*Hospital Infanta Margarita* in Cabra, Córdoba). This indicates a low incidence, as does a previous study by Palazón Sánchez et al. [1].

Furthermore, we believe that the use of benzodiazepine antagonist drugs (flumazenil) and opiate antagonists (naloxone) in emergency departments means that many patients do not require intensive care.

It is, furthermore, a pathology with a low rate of mortality. Our study showed an ICU mortality of 5.9% and a hospital mortality of 6.7%. These figures are in agreement with the published literature [5, 7, 8].

However, this should not lead us to the underestimation of the severity of these patients. We all hear in the news about fatal cases of poisoning which, in some cases, were not diagnosed in time to save the patient's life. This in turn contributes to the fact that it is a disease with multiple causes, and each case has a different clinical picture. So, poisoning may often go undetected. Severe cases which present with shock and multiorgan failure (as with poisoning by methanol) cannot be readily diagnosed and may be confused with other entities such as sepsis. This can put the patient's life at risk because they do not receive the appropriate treatment.

The heterogeneous nature of the clinical picture of poisoning is also important in considering severity. Evolution, mortality, and prognosis are very different in each case and depend principally on the poison. In fact, our study shows one group of patients with a very high rate of mortality, those with caustic poisoning, where mortality was comparable to that observed in other studies on this type of patient, in whom morbidity and observed complications are very high, and mortality depends on many factors such as age, the type of caustic, the amount ingested, and others [18–22].

With regard to the performance of the prognostic indices which are regularly used in the ICU, that is, APACHE-II, APACHE-III, and SAPS-3, in relation to these patients, this study is part of a line of work which we have been carrying out for some time that focuses on the development and evaluation of prognostic systems for patients in the ICU. In recent years, we have evaluated the usefulness of SAPS-2 and SOFA [23] and how SAPS-3 and APACHE-III are applied in Spain [9, 10].

Our work shows some important discrepancies between the mortality predicted by SAPS-3 and the observed mortality. There were statistically significant differences. However, we observed no discrepancies with the two versions of the APACHE system and found the observed and predicted mortality rates to be very similar.

Really, as far as the SAPS models are concerned, investigators have recognized that the original SAPS-2 model was far too pessimistic in estimating the mortality rates of patients suffering from drug or alcohol intoxication [24].

In the same line, it is necessary to mention an important French study whose objective was to improve the Simplified Acute Physiology Score (SAPS) II for mortality prediction in ICUs, thereby improving standardized mortality ratio estimates [25]. Drug-overdose was observed in 11.86% of 77.490 admissions from 106 French ICUs. Calibration and discrimination were determined for the original SAPS II, a customized SAPS II, and an expanded SAPS II developed in the training set by adding six admission variables: age, sex, length of pre-ICU hospital stay, patient location before ICU, clinical category, and whether drug overdose was present. The training set was used for internal validation and the validation set for external validation. The expanded SAPS II model exhibited excellent calibration.

An analysis into the causes of these discrepancies showed that these patients present with conditions which are associated with high scores in the calculation of these prognostic indices, when compared with other patients; that is, they are nonsurgical patients and are therefore assigned a higher score than those admitted for elective surgery. They are admitted as urgent or emergency patients and are, therefore, once again, assigned a higher score than scheduled admissions. Furthermore, many of them are admitted in a state of coma, for which they score 4 points.

In our study, 72.5% of patients had a GCS ≤8 points upon admission, and this led to higher scoring on SAPS-3 and the two versions of APACHE. However, this can lead to mistakes, as the course of these patients is very different to others with a low Glasgow score and a structural cerebral pathology (such as patients with head trauma or severe brain haemorrhage). Patients with a structural cerebral pathology have a high rate of mortality, which is not the case with patients admitted to the ICU for poisoning. Even though patients with a focal neurological deficit are assigned a correction factor of 10 and not 4 (as is the case with those admitted in a coma), this does not seem to be sufficient, and this correction factor does not make up for the difference in mortality between those patients admitted with poisoning and those admitted with a structural cerebral pathology.

This low GCS score also explains the high rate of patients who required mechanical ventilation on admission (69.7%); though observing their subsequent clinical course and their short stay, we know that their development is satisfactory, and the majority of these patients recover an adequate level of consciousness and mechanical ventilation can be withdrawn early.

In the SAPS-3 cohort of 19,577 patients, there were only 224 cases with drug overdose (1.14%) and 229 of other intoxications (1.17%). When a score developed in a group of patients where a specific illness is poorly represented is applied to a new population with a high incidence of the specific illness (external validation studies) indeed it will have a poor performance [26]. This fact could partly explain the discrepancies between the mortality predicted by SAPS-3 and the observed mortality in our study in this type of patients. The bad observed correlation in our study in poisoning patients cannot be generalized to other ICU patients.

Continuing with the analysis of the causes of the observed discrepancies, we must consider that the APACHE-II and APACHE-III systems include in their list of reasons for admission to ICU drug overdose, and these patients are given a specific coefficient, which is not the case with SAPS-3. In the APACHE system, the diagnostic category weight for “drug overdose” is the most negative value for nonoperative diagnoses, due to the well-known good outcome of this patient population. On the other hand, patients admitted for caustic intoxication received a diagnostic category weight suitable for other symptoms (i.e., respiratory failure or major organ system involved as the principal reason for admission). This appears to be the reason why SAPS-3 overestimates the severity of patients admitted with poisoning, unlike the APACHE systems. For this reason, the APACHE systems are useful in terms of providing information to the medical staff who attended to these patients and to the families about the risk of death, while SAPS-3 does not provide real information about the severity of these patients, but it rather overestimates it.

With regard to the limitations of our work, the number of patients included is quite small for such a kind of analysis. Usually, validation studies require large samples to obtain reliable and statistically significant conclusions. With small differences, it is necessary to assess a large sample to obtain statistically significant results. Our study found significant differences in the observed mortality predicted by SAPS-3, and although the number of patients was small, it was sufficient to draw conclusions based on findings which were statistically significant. We can affirm that the SAPS-3 overestimates mortality in these patients, with statistically significant differences. In the case of APACHE-II and APACHE-III, a larger sample is necessary because the differences between the observed and expected mortality were small and were not statistically significant. The small number of patients included in “caustic” diagnostic category (*n* = 11) explains the OR's broad confidence interval (OR: 560.34 (11.64–26973.83)). Our study shows that the risk of dying of these patients is greater than the other patients included in the study (other types of poisoning). But it is not possible to specify a clinical useful confidence interval. Numerous previous studies, with smaller samples than ours, have shown statistically significant differences and their conclusions have been accepted. This is common in mechanical ventilation and respiratory pathology studies. Our group has published studies [27–29] with a smaller number of subjects, but this was sufficient to find differences and come to valid conclusions, which have been confirmed in subsequent studies.

Other possible limitation is the different profile of poisoning that can be modified according to the country. A recently published study on Iranian patients admitted for poisoning presented an intra-ICU mortality of 21.5% [30]. This rate is higher than that found in our study, but these authors agreed with the usefulness of APACHE-II as a predictor system. This difference in mortality may be due to different types of poisoning. Our article shows the highest mortality rate of patients with caustic poisoning.

Our results are similar to those of other recently published studies, such as Brandenburg et al. [7], who had a far larger study sample (7331 patients) and observed a low rate of incidence and mortality. Also, Liisanantti et al. [8] included 255 patients admitted to the ICU in 28 hospitals over a period of six years with results very similar to our own.

There are, however, few publications within the field of intensive care about poisoning. For this reason, our study is important as we intend to throw some light on the current incidence and mortality of this pathology, in spite of the limitations mentioned above.

In conclusion, admission to ICU for poisoning is not common. There is frequently in these cases an impact on the level of consciousness and these patients often need mechanical ventilation upon admission. Mortality among patients admitted for the ingestion of caustics is far higher than among patients admitted for other types of poisoning. The APACHE-II and APACHE-III systems provide adequate predictions of mortality among these patients, unlike SAPS-3, which is not useful in evaluating their mortality or in providing information about the risk of death to family members or medical practitioners attending to these patients. SAPS-3 tends to overestimate mortality in these cases.

## Figures and Tables

**Table 1:** Basic demographic data.

Age	44.42 ± 13.85 [36 (44,53)]
Type of poisoning^*∗*^	
Drug overdose	92 (77.3%)
Alcohol	20 (16.8%)
Caustics	11 (9.2%)
Attempted suicide	78.3%
Glasgow Coma Scale at admission	8.39 ± 4.51 [4 (7,13)]
Mechanical ventilation at admission	69.7%
APACHE-II	16.29 ± 7.17 [11 (16,21)]
APACHE-III	47.68 ± 26.33 [28 (48,69)]
SAPS-3	54.17 ± 11.33 [46 (53,60)]
Predicted mortality by SAPS-3	26.98%
(general equation)	
Predicted mortality by SAPS-3	27.78%
(Southern Europe, Mediterranean equation)	
Predicted mortality by APACHE-II	7.57%
Predicted mortality by APACHE-III	8.15%
ICU mortality	5.9%
Hospital mortality	6.7%

^*∗*^2 patients (1.7%) had ingested drug overdose and caustics. 9 patients (7.6%) had ingested drug overdose and alcohol.

*Note*. Quantitative variables are expressed as mean ± standard deviation and median with interquartile range in brackets.

**Table:** (a) Relationship between mortality and quantitative variables

	Survivors	Nonsurvivors	*p*
	(*n* = 111)	(*n* = 8)	
Age	43.35 ± 13.49 [35 (44,52)]	59 ± 10.64 [51 (59,68)]	0.002
GCS at admission	8.30 ± 4.83 [4 (7,13)]	9.83 ± 5.04 [4 (10,14)]	0.503
APACHE II	15.53 ± 6.77 [11 (16,20)]	26.87 ± 3.44 [24 (28,29)]	<0.001
APACHE III	44.56 ± 24.21 [27 (43,65)]	91 ± 12.07 [81 (91,103)]	<0.001
SAPS-3	53.51 ± 10.6 [46 (53,69)]	63.25 ± 17.16 [48 (59.78)]	0.167
Predicted mortality by SAPS-3 (general equation)	25.9 ± 27.25 [12 (22,34)]	41.89 ± 29.39 [14 (34,71)]	0.167
Predicted mortality by SAPS-3 (Southern Europe equation)	26.76 ± 16.83 [13 (24,35)]	41.92 ± 28.45 [15 (35,69)]	0.167
Predicted mortality by APACHE-II	3.49 ± 9.57 [0.6 (1,3)]	64.11 ± 15.66 [48 (68,77)]	<0.001
Predicted mortality by APACHE-III	5.29 ± 8.55 [0.9 (3,7)]	47.9 ± 16.54 [33 (53,60)]	<0.001

Quantitative variables are expressed as mean ± standard deviation and median with interquartile range in brackets.

**Table:** (b) Relationship between mortality and qualitative variables

	Mortality	*p*
Attempted suicide		0.508
Yes (*n* = 93)	7 (7.5%)	
No (*n* = 26)	1 (3.8%)	
Mechanical ventilation at admission		0.054
Yes (*n* = 83)	8 (9.6%)	
No (*n* = 36)	0 (0%)	
Type of poisoning		
Drug overdose		<0.001
Yes (*n* = 92)	1 (1.1%)	
No (*n* = 27)	7 (9.9%)	
Alcohol		0.180
Yes (*n* = 20)	0 (0%)	
No (*n* = 99)	8 (8.1%)	
Caustics		<0.001
Yes (*n* = 11)	6 (54.5%)	
No (*n* = 108)	2 (1.9%)	

**Table 3:** Relationship between ingestion of caustics and other variables.

	Caustics	Noncaustics	*p*
	(*n* = 11)	(*n* = 108)	
Age	58.91 ± 14.24 [54 (60,69)]	42.94 ± 12.92 [35 (43,51)]	<0.001
Attempted suicide	100%	75.9%	0.066
GCS at admission	12.82 ± 2.93 [11 (14,15)]	7.94 ± 4.41 [4 (7,12)]	0.001
Mechanical ventilation at admission	90.9%	67.6%	0.109
APACHE-II	20.09 ± 9.09 [11 (21,30)]	15.91 ± 6.88 [11 (16,21)]	0.112
APACHE-III	63.36 ± 33.69 [29 (72,95)]	46.08 ± 25.07 [27 (47,65)]	0.105
SAPS-3	54.73 ± 14.24 [47 (51,57)]	54.11 ± 11.15 [46 (53,60)]	0.676
Predicted mortality by SAPS-3 (general equation)	26.28 + 22.34 [13 (19,29)]	27.05 + 18.32 [12 (23,36)]	0.676
Predicted mortality by SAPS-3 (Southern Europe equation)	27.13 + 21.38 [14 (20,31)]	27.85 + 17.81 [13 (24,37)]	0.676
Predicted mortality by APACHE-II	47.68 + 30.01 [15 (51,77)]	3.48 + 10.03 [3 (6,11)]	<0.001
Predicted mortality by APACHE-III	29.53 + 26.36 [3 (9,54)]	5.97 + 10.14 [6 (8,31)]	0.003
ICU mortality	54.5%	0.9%	<0.001
Hospital mortality	54.5%	1.9%	<0.001

Quantitative variables are expressed as mean ± standard deviation and median with interquartile range in brackets.

**Table:** (a) Performance of the SAPS-3 score. Goodness of fit of general SAPS-3 model by *H*-Hosmer-Lemeshow statistic

Probability of death^*∗*^	Number of cases	Number of deaths		Number of survivors	
		Observed	Predicted	Observed	Predicted
≤0.2	50	3	6.5	47	44.45
0.2–0.4	45	1	12.85	44	32.15
0.4–0.6	13	1	6.08	12	6.92
0.6–0.8	10	2	6.76	8	3.24
>0.8	1	1	0.86	0	0.14

^*∗*^Probability of death based in general equation *H* = 35.10; DF 3; *p* < 0.001.

**Table:** (b) Performance of the SAPS-3 score. Goodness of fit of Southern Europe, Mediterranean countries SAPS-3 model by *H*-Hosmer-Lemeshow statistic

Probability of death^*∗*^	Number of cases	Number of deaths		Number of survivors	
		Observed	Predicted	Observed	Predicted
≤0.2	43	3	4.62	40	38.38
0.2–0.4	52	1	14.86	51	37.14
0.4–0.6	14	1	6.72	13	7.28
0.6–0.8	9	2	6.03	7	2.97
>0.8	1	1	0.83	0	0.97

^*∗*^Probability of death based in Southern Europe and Mediterranean countries *H* = 36.47; DF 3; *p* < 0.001.

**Table:** (a) Performance of the APACHE-II score. Goodness of fit of APACHE-II model by *C*-Hosmer-Lemeshow statistic

Probability of death^*∗*^	Number of cases	Number of deaths		Number of survivors	
		Observed	Predicted	Observed	Predicted
≤0.051	24	0	0.08	24	23.92
0.051–0.0106	24	0	0.19	24	23.81
0.0106–0.0189	24	0	0.33	24	23.67
0.0189–0.0384	24	0	0.62	24	23.38
>0.0384	23	8	7.78	15	15.22

^*∗*^Probability of death based in APACHE-II equation *C* = 1.2563; DF 3; nonstatistical significance.

**Table:** (b) Performance of the APACHE-III score. Goodness of fit of APACHE-III model by *C*-Hosmer-Lemeshow statistic

Probability of death^*∗*^	Number of cases	Number of deaths		Number of survivors	
		Observed	Predicted	Observed	Predicted
≤0.078	24	0	0.10	24	23.90
0.078–0.027	24	0	0.33	24	23.67
0.027–0.051	24	0	0.84	24	23.16
0.051–0.0953	24	0	7.72	24	22.28
>0.0953	23	8	6.71	15	16.29

^*∗*^Probability of death based in APACHE-III equation *C* = 3.51; DF 3; nonstatistical significance.

## Abbreviations

APACHE: Acute Physiology and Chronic Health Evaluation
GCS: Glasgow Coma Scale
ICU: Intensive care unit
OR: Odds ratio
SAPS: Simplified acute physiology score
SOFA: Sepsis-related Organ Failure Assessment.
